# Pharmacogenomics Implications of Using Herbal Medicinal Plants on African Populations in Health Transition

**DOI:** 10.3390/ph8030637

**Published:** 2015-09-21

**Authors:** Nicholas E. Thomford, Kevin Dzobo, Denis Chopera, Ambroise Wonkam, Michelle Skelton, Dee Blackhurst, Shadreck Chirikure, Collet Dandara

**Affiliations:** 1 Division of Human Genetics, Department of Pathology & Institute of Infectious Diseases and Molecular Medicine, Faculty of Health Sciences, University of Cape Town, Cape Town, Observatory 7925, South African; E-Mails: THMNIC023@myuct.ac.za (N.E.T.); ambroise.wonkam@uct.ac.za (A.W.); michelle.skelton@uct.ac.za (M.S.); 2 School of Medical Sciences, University of Cape Coast, Cape Coast, Ghana; 3 ICGEB, Cape Town component, Faculty of Health Sciences, University of Cape Town, Cape Town, Observatory 7925, South Africa; E-Mail: kd.dzobo@uct.ac.za; 4 Division of Medical Biochemistry, Faculty of Health Sciences, University of Cape Town, Cape Town, Observatory 7925, South Africa; 5 Division of Medical Virology, University of Cape Town, Cape Town, Observatory 7925, South Africa; E-Mail: denis.chopera@uct.ac.za; 6 Division of Chemical Pathology, University of Cape Town, Cape Town, Observatory 7925, South Africa; E-Mail: dee.blackhurst@uct.ac.za; 7 Department of Archaeology, University of Cape Town, Cape Town, Rondebosch 7701, South Africa; E-Mail: shadreck.chirikure@uct.ac.za

**Keywords:** herbal medicine, conventional medicine, CYP450, malaria, HIV/AIDS, hypertension, bleeding disorders, pharmacogenetics

## Abstract

The most accessible points of call for most African populations with respect to primary health care are traditional health systems that include spiritual, religious, and herbal medicine. This review focusses only on the use of herbal medicines. Most African people accept herbal medicines as generally safe with no serious adverse effects. However, the overlap between conventional medicine and herbal medicine is a reality among countries in health systems transition. Patients often simultaneously seek treatment from both conventional and traditional health systems for the same condition. Commonly encountered conditions/diseases include malaria, HIV/AIDS, hypertension, tuberculosis, and bleeding disorders. It is therefore imperative to understand the modes of interaction between different drugs from conventional and traditional health care systems when used in treatment combinations. Both conventional and traditional drug entities are metabolized by the same enzyme systems in the human body, resulting in both pharmacokinetics and pharmacodynamics interactions, whose properties remain unknown/unquantified. Thus, it is important that profiles of interaction between different herbal and conventional medicines be evaluated. This review evaluates herbal and conventional drugs in a few African countries and their potential interaction at the pharmacogenomics level.

## 1. Introduction

It has been observed that an increasing number of people in developed and developing countries use herbal products for both preventive and therapeutic purposes. Often the use of herbal medicines is associated with attenuation of side-effects produced from therapeutic drugs [1]. For centuries humanity has used plants for medicinal purposes and over the last few decades there has been a most remarkable revival of herbal medicine. This has been partly due to the elusive cure for HIV/AIDS that has prompted many countries to refocus their attention on herbal medicinal plants, with increased funding being poured into related research areas. Herbal medicines are used for the treatment of many ailments, including immune and psychiatric disorders, microbial and viral infections, non-communicable diseases such as cancer, malaria, and injuries, and reproductive health issues such as infertility. Some of the factors that facilitate high usage of herbal medicines include their local abundance, cultural significance, history of known efficacy, and, most importantly, inexpensive procurement compared to conventional pharmaceuticals [2]. It is estimated that 30%–50% of all drugs on the market are either derived from or their design was inspired by herbal medicinal plants [3]. The World Health Organization (WHO) estimates that 80% of Asian and African populations rely on traditional medicine for their primary health care needs and in the developed countries 70%–80% of the population use some form of complementary and alternative medicine at some stage [4].

The use of medicinal herbal plants is one of the oldest known forms of therapy, which has evolved in such a way that each culture, country, or region has created its own pharmacy of locally grown medicinal plants. Herbal medicinal products are easily accessible. They are found in informal markets, pharmacies, health-food stores, and even online [5]. This abundance has, however, brought with it the issue of “fake” herbals which find their way onto the market alongside real ones. As herbal remedies are considered “pure and natural” there is a belief that they are also “safe and harmless” [6]. Although herbal medicines are from natural sources, some naively believe that they are always non-toxic [7] and that they are always complications-free after use. And yet, there are several stories of patients who developed complications and even died after taking some herbal medicines [8]. In fact, in some parts of Africa, it is common practice that herbal traditional healers or doctors withhold some of the herbal medicines from their patients and only dose them under their strict supervision because of known toxicities should overdoses occur. However, most of the herbals are prescribed as “take home” treatments making it easy for patients to “overdose” themselves.

In terms of pharmacogenomics, it is known that medicinal herbal plants are metabolized by drug metabolizing enzymes (DMEs). This has several implications for their therapeutic efficacy. Firstly, DMEs are encoded for by polymorphic genes which have a potential to affect the way these compounds are metabolized. The variation in DME genes results in variant enzymes presenting with altered activity or abolished activity [9,10]. Thus, in a population with individuals carrying a myriad of polymorphic DME genes, a huge variability exists in the way individuals respond to any of the herbal medicines taken. This leads to classification of people into different drug response phenotypes, which includes poor metabolizers, extensive metabolizers, and ultra-rapid metabolizers, with respect to specific drugs, which then influences the drug’s physiological effects [11]. It is therefore necessary to take into account the pharmacogenetics effects of DMEs when herbal medicinal plants are used as a source of therapy. Besides being metabolized, herbal medicinal drugs are also able to affect the expression of some of the DMEs, either through inhibition or induction [12,13].

The same enzyme system that metabolizes herbal medicinal compounds is also responsible for the metabolism of conventional drugs. This has huge implications among patients who intermittently make use of both health care systems. In some cases, herbal medicinal plants induce DMEs that metabolize particular conventional drugs such that, when given together, the conventional drug does not reach therapeutic levels because of the increased abundance of the DME [14]. On the other hand, herbal medicinal drugs could present with inhibitory effects preventing/delaying the removal of co-administered conventional drugs and resulting in them reaching toxic levels. According to the World Health Organization, Africa is burdened with increasing communicable and non-communicable diseases that pose many challenges on already poorly resourced health systems [15]. Medicinal herbal plants present an alternative for combating these diseases, with the aim of reducing their associated morbidity and mortality. However, the pharmacogenetics implications of the use of these herbal plants have not been properly assessed.

In this article, we report on the use of medicinal herbal plants in the era of HIV/AIDS, malaria, and increasing non-communicable diseases, and their pharmacogenetics implications, focusing on Southern and West African populations. Literature related to herbal medicine use and genetic polymorphisms for DMEs known to metabolize these was evaluated.

We performed a bibliometric analysis related to herbal medicine use and genetic polymorphisms for DMEs known to metabolize various drugs to evaluate the use of medicinal herbal plants in the era of HIV, malaria, and some non-communicable diseases, and their pharmacogenetics implications. The study focused mostly on data available from a selected group of Southern and West African countries and these countries were chosen mostly due to accessibility.

## 2. Search Methods for Relevant Literature

The literature for this review was accessed through databases such as PubMed/Medline and Google Scholar. The main words/phrases used in the search were: drug-drug interactions, drug-herb interactions, herb-herb interactions, herbal medicines, malaria, HIV/AIDS, TB, hypertension, bleeding disorders, herbal treatment, pharmacogenetics/pharmacogenomics, and cytochrome P450. Individual words or combinations were narrowed down by emphasizing “Africa”, then lastly, Southern Africa and West Africa, separately. Only articles that could be accessed in full format were used. The review was limited to the disorders mentioned above and herbal plants reported to be commonly used by Southern and West African populations. We acknowledge that the review is not exhaustive of all the published sources. However, the wide-ranging nature of the search criteria and phrases used in the search enabled us to discuss in detail the implications of pharmacogenomics in the use of herbal medicinal plants.

## 3. Discussion of Pharmacogenomics Implications on the Co-use of Conventional and Herbal Medicines

### 3.1. Brief Overview

Developing countries are notoriously affected by many health challenges, especially “diseases of poverty”, such as waterborne diseases, viral infections, such as HIV/AIDS and influenza, malaria, and an emerging pandemic of non-communicable diseases. The intersection of all these disease conditions means that, at any time, in any one patient, there is a high likelihood of receiving drugs that are metabolized by the same enzyme. This has huge implications, since this leads either to sub-therapeutic levels or high concentrations that reach toxic levels. Table 1 shows a list of some drugs used for the treatment of commonly encountered health challenges, highlighting the overlapping enzyme system.

Adding medicinal herbal plants to this already complicated picture further adds to major health issues. A close look at Southern, Eastern, Central, and West Africa shows that there are certain common herbal medicinal drugs that are prescribed for the treatment of commonly occurring conditions, such as malaria, hypertension, and HIV/AIDS [16,17]. Some of the medicinal drugs used in these different regions either belong to the same plant species or are derived from closely related plant species. Examples of these include *Moringa pterygosperma* and *Moringa oleifera*, which have a wide use in sub Saharan Africa Many studies have evaluated the metabolism of some of these medicinal drugs with respect to the enzymes that are involved. The main indication is that there is an overlap in the DMEs that are involved in the metabolism of each of the herbal compounds, just as in the case of conventional drugs. For both health systems (herbal and conventional), the polymorphisms in DMEs affect their activities and, therefore, their disposition. It is important to note that genetic variants of DMEs exhibit both qualitative and quantitative differences between different racial and ethnic populations. Indeed, *CYP2D6* provides a very good example, where certain variants are only found in particular populations, for example: CYP2D6*17 among black Africans, CYP2D6*10 among Asians, and CYP2D6*2N reported in most populations but at different frequencies [18]. Among patients using both the conventional and traditional drug systems, the issues to contend with include drug-drug, herb-drug, herb-herb interactions, and genetic polymorphisms in genes coding for DMEs. Below we evaluate specific examples of diseases/conditions and how they are managed by the different regimens, be it conventional or traditional herbal medicines.

**Table 1:** Common drugs prescribed for malaria, hypertension, and HIV and their metabolizing enzymes.

Disease	Drug	Herbals	DMEs	Ref.
Malaria	Chloroquine Artesunate Amodiaquine Artemisinin Quinine Lumefantrine/Artemether	*Phyllanthus amarus*; *Momordica charantia*	CYP2C8,CYP2D6, CYP3A4	[19]
			CYP2A6	[20]
			CYP2C8	
			CYP3A4	
			CYP3A4	[20]
			CYP3A4/5, CYP2B6, CYP2C9, CYP2C19	[21]
HIV	Efavirenz Lopinavir Zidovudine Stavudine* Ritonavir Nevirapine Emtricitabine* Tenofovir	*Sutherlandia frutescens* *Hypoxis hemerocallidea* *Tridax procumbens*	CYP2B6	[22]
			CYP3A4	[23]
			UDPGT	[24]
			NA	
			CYP3A4,CYP2D6	[25]
			CYP3A4	[26]
			NA	[27]
			Esterases	
Hypertension	Atenolol * Lisinopril * Losartan Nifedipine Verapamil Amlodipine	*Lactuca taraxicifolia*	N/A	[28]
			N/A	[28]
			CYP2C9,CYP3A4	[28,29]
			CYP3A4	[28,30]
			CYP3A4	[31]
			CYP3A4	[32]

*N/A = not metabolized by cytochrome P450 or transformed; DME = drug metabolizing enzyme.

### 3.2. A Focus on Malaria

#### 3.2.1. Herbal Medicinal Herbal Plants Used in the Management and Treatment of Malaria

Malaria is one of the leading causes of morbidity and mortality with 124–283 million estimated cases per year, resulting in an estimated 367,000–755,000 fatalities [19]. Conventional drug development has often neglected malaria such that most of the drug discoveries emanated from accidents or drug-repurposing [33]. The use of plant-derived drugs for the treatment of malaria has a long and successful tradition, with a litany of many plants being indicated for the treatment of malaria, with some of the compounds providing good sources for the detection of active components with novel anti-plasmodial activities [34]. The first antimalarial drug, quinine, was isolated from the bark of Cinchona species found in South America (*Rubiaceae*) in 1820 and is one of the oldest and most important antimalarial drugs, which is still in use today [35]. In the 1940s, another antimalarial drug, chloroquine, was synthesized and is also currently in use for the treatment of malaria [36]. In Ghana there is rich literature on the use of plants for malaria treatment by various local communities [37,38]. Some of the anti-malarial medicinal plants used in this country include *Alstoonei boonei* [39], *Morinda lucida* [40], *Cryptolepis sanguinolenta* [37], *Hyptis spp* [41], *Moringa oleifera*, and *Azadirachta indica* [38].

The evidence from South African traditional healers also demonstrates a wide use of herbal medicine in combating malaria [42,43]. For example, there is extensive utilization of *Mimusops caffra*, *Mimusops obtusifolia*, and *Hypoxis colchicifolia* for the management of malaria [44]. Other commonly used species include *Gardenia* *thunbergia*, *Siphonochilus aethiopicus*, *Schotia brachypetala*, *Acorus calamus*, *Withania somnifera*, *Elaeodendron transvalense*, *Hypoxis hemerocallidea*, *Vernonia* *adoensis*, and *Acanthospermum australe*, which are frequently utilized by Zulu traditional medicine practitioners [45]. Although all these plants have been commonly used by traditional healers, their efficacy has not been extensively studied or scientifically validated. Maybe, this is one of the problems with conventional medicine that, as we seek to scientifically validate the efficacy of herbal medicines, we take a reductionist approach, wanting to work with extracted components and not mixtures. *Cassia sieberiana* and *Vernonia amygdalina* have at least been scientifically shown to exhibit antimalarial properties, further justifying their extensive utilization for malaria prevention [46].

#### 3.2.2. Conventional Drugs Used in the Management of Malaria and the Pharmacogenetics Landscape

The use of herbal medicine has been the mainstay for the clinical control of malaria for hundreds of years. For example, artemisinin rich teas were introduced in China 1500 years ago [47], while in the 17th century South America tree barks containing quinine were exploited. It was only in the 20th century when synthetic and semi-synthetic compounds started to be developed. The pharmacogenetics of antimalarial herbs is, however, poorly known, although its application might be critical in the optimization of malaria treatment [48].

It has recently been demonstrated that host genetic variation in DMEs influences the selection of *Plasmodium falciparum* drug-resistance among Burkinabe patients [49]. Cytochrome P450 2C8 (CYP2C8), a polymorphic enzyme that mainly contributes to the hepatic metabolism of amodiaquine (AQ) and chloroquine (CQ), presents with several genetic variants (e.g., CYP2C8*2) that are associated with decreased CYP2C8 enzyme activity, which affects metabolism of CYP2C8 substrate drugs, thereby leading to variability in the removal of malaria causing parasites in the infected hosts [50]. *CYP2C8* polymorphic variants show qualitative and quantitative differences between different populations, for example, allele CYP2C8*2 is the most common variant in Africans, associated with the poor metabolizer phenotype (PM), whereas CYP2C8*3 is rare among Africans [51]. While the effects of genetic polymorphisms for conventional drugs used in the treatment of malaria seems to be well studied, there is no data on the effects of the various antimalarial herbal medicines on CYP2C8 or, *vice-versa*, there is no data on which herbal medicines are substrates for CYP2C8, information that is important in understanding the differential effects of herbal medicine among populations that are in different geographical locations or of different racial extraction. 

### 3.3. HIV/AIDS Management

#### 3.3.1. Scrounging for a Cure: the Use of Medicinal Herbal Plants in HIV/AIDS Management

An HIV cure has been elusive for the last 30 years and all current drugs have been effective only in reducing the viral load but not in eliminating the virus completely. Several antiretroviral drugs are on the market and their mode of action involves combination therapy. Despite the availability of several pharmaceutical drugs, their cost and associated side-effects makes the majority of African populations unable to afford or continue on these treatments on a consistent and/or sustainable basis [52]. Access, especially to second-line therapies, is often well beyond the means of most patients [53], making it difficult to meet the World Health Organization’s (WHO) plans to have 15 million persons on antiretroviral drugs by 2015 [54]. The virus resistance and toxicity [53] associated with some of these drugs makes it difficult for patients to enjoy quality of life while taking them. Looking at all these challenges faced by conventional medical care, it has been seen that there is a recent boom in the use of traditional medicine systems, especially in sub-Saharan Africa (SSA) where HIV/AIDS is endemic. The lack of suitable vaccines has also propelled the use of herbal medicinal plants. Thus, we are at a juncture, where in search of good health, in many regions of Africa and elsewhere, where people start using medicine from the two health systems simultaneously or sequentially, as noted by King & Homsy [55], resulting in a kind of medical pluralism.

HIV/AIDS research has received a lot of attention, especially vaccine development and, lately, drug discovery and development. The failure to get a cure has increased the attention to herbal medicinal plants. Some African indigenous plants have been shown to possess antiretroviral activity using *in vitro* experiments [56,57]. The herbals that have shown promise are being tested as treatments for HIV- related infections. The use of traditional medicine and natural health products is widespread among those living with HIV infection [58]. Ethnobotanical studies conducted in different African countries, for example, Uganda [59], Tanzania [60], Namibia [61], and Cameroon [62], show that traditional healers and lay-people extensively utilize medicinal plants to manage the effects of HIV/AIDS. Two principal African herbal compounds used for HIV/AIDS management in sub-Saharan Africa (SSA) include *Hypoxis* *hemerocallidea* (common name: African potato) and *Sutherlandia frutescens*. These two herbal remedies are currently recommended by the South African Ministry of Health for HIV management [16]. The 14 member states of the Southern African Development Community (SADC), namely, Angola, Botswana, Democratic Republic of Congo, Lesotho, Malawi, Mauritius, Mozambique, Namibia, Seychelles, South Africa, Swaziland, Tanzania, Zambia, and Zimbabwe, also support their use [16].

A cross-sectional survey has indicated the use of medicinal herbal plants in the management of HIV/AIDS among patients in Ghana [63]. Two plants, *Betula alba* and *Sutherlandia* *frutescens*, have found their way into herbal clinics for the management of HIV/AIDS due to their immunostimulatory and antimicrobial properties [17]. *Anona senegalense* is an herbal plant that has been acknowledged to be used for HIV/AIDS treatment by the people of the Northern part of Ghana [38]. As part of an ethnobotanical research, it was noted that *Cassia abbreviate,* *Cassia sieberiana, Coccinia rehmannii, Combretum albopanctum*, *Combretum imberbe*, *Diospyros lycioides*, *Pavetta harborii*, *Plumbago zeylanica*, *Spermacoce deserti*, and *Ximenia caffra* are some of the common medicinal plants used by traditional healers in the management of HIV/AIDS in Botswana [64]. However, it is worth noting that the partial benefit attributed to some traditional medicines in HIV/AIDS management could be due to their immune-boosting activity rather than a direct inhibitory effect on viral replication.

#### 3.3.2. FDA Approved ARVs and Pharmacogenetics Landscape

The use of combinations of antiretroviral drugs to provide potent antiretroviral therapy (ART) has dramatically impacted the morbidity and mortality due to HIV/AIDS [65]. Despite this revelation, clinicians are increasingly faced with challenges in the selection and management of antiretroviral therapy regimens, including the choice of the most efficacious drugs that have the lowest toxicity and do not interact with other drugs used for commonly occurring conditions. Although the introduction of viral genotyping and combination-therapy pharmacokinetic data has provided some guidance, the investigation of host genetic factors that impact both the efficacy and toxicity of ART may also aid in selecting the best regimen for individual patients [66]. African populations are among the most genetically diverse [67] and possibly present with a wide range of pharmacogenomics profiles that need to be considered for each drug or class of drugs [68].

Several pharmacogenetics studies on HIV/AIDS cohorts on EFV-containing regimens have shown that the *CYP2B6 c.516G>T* SNP affects plasma EFV levels. This has been reported among Ghanaians [69], South Africans [18], Zimbabweans [70], Ethiopians and Tanzanians [71], and among Caucasians [72]. Several other variants, such as *CYP2B6 c.983T>C* [73] and *CYP2B6 g.15582C>T* [74], have also been shown to play a significant role in plasma EFV concentration. Genetic variation in *CYP2A6*, *NR1I3* (coding for constitutive androstane receptor-CAR), and UGT2B7 has also been shown to be associated with significantly elevated efavirenz concentrations among HIV/AIDS patients. For *NR1I3*, the *NR1I3 c.540C>T* SNP seems to play a significant role in plasma efavirenz concentration [75,76]. There is an imperative need to use this knowledge on the known DMEs that affect ARV drugs in adding value to herbal medicines. For example, very little information is available on how and which herbal medicines affect the activities of enzymes such as CYP2B6. For example, *in vitro* inhibition studies using heterologously expressed CYP2B6, incubated with its probe-substrates in the presence of specific herbal compounds, may assist in evaluating if there is any inhibition of activity. In order to be able to identify specific therapeutically active components (e.g., phytochemicals) in herbal extracts, various computational tools and models, such as pharmacophore modelling, virtual screening, docking, and neural networking [77], can be employed. Whatever components are identified could become candidates for possible rational drug design. Identifying profiles of differential RNA, protein expression, and metabolites in cells or patients exposed to particular herbal medicines could take advantage of the advanced “Omics” technologies, such as genomics, proteomics, and metabolomics [78] to ultimately come up with response profiles. These profiles may be used for assessments of efficacy and safety, further validating the utility of herbal compounds as is done for conventional medicine.

### 3.4. Hypertension Treatment

#### 3.4.1. The Use of Medicinal Herbal Plants in Hypertension Management and Treatment 

The burden of hypertension (HTN) in Africa is widely reported, with the World Economic Forum acknowledging its effects on productivity [79]. Hypertension is the most frequently observed risk factor for cardiovascular diseases (CVD) in both urban and rural communities in sub-Sahara Africa (SSA) and will contribute to the growing burden of CVD [80]. Traditional herbal medicine may have significant impact on HTN treatment and control in sub-Saharan Africa [81]. Traditional medicine use is high among adults in SSA with a prevalence ranging from 38.5%–90% [82]. Traditional herbal medicine is commonly used around the world for both cardiovascular disease in general [83] and hypertension specifically [84].

According to Ziblim *et al.* [38], in the Northern parts of Ghana, medicinal herbal plants such as *Parkia biglobos* and *Moringa oleifera* are used in the management of hypertension. As mentioned earlier, different regions or geographical settings have their own plants that they use to manage common conditions they encounter. For example, *Tetrapleura tetraptera* and *Alstonia boonei* are used by the Akan people, who are mostly found in the southern and coastal towns of Ghana, for managing hypertension [85], and *Launaea taraxacifolia* is used in the treatment of hyperlipidemia, which is a risk factor for hypertension [86] and stroke [87].

In Botswana, *Hydnora johannis*, *Cassine transvaalensis*, *Cassia abbreviate*, and *Elephantorrhiza* *goetzei* [88], and *Myrothamnus flabellifolius* [89], are indicated for the management of high blood pressure conditions, whereas, *Agathosma betulina* has been used among South African population groups as an effective diuretic and anti-inflammatory agent [90]. There are also reports that *Allium sativum* has been reported to have hypotensive effects. A compilation of medicinal plants in South Africa has also listed *Aloe ferox* and *Hoodia gordonii* as plants used in managing hypertension in South Africa.

#### 3.4.2. Anti-Hypertensive Drugs and Related Pharmacogenetics Observations

High blood pressure (BP) is the most common single risk factor for cardiovascular-related events and deaths worldwide [91]. In a recent review, it was observed that in the last 10 years incidence of systolic BP has risen more in most of Africa, except the east, than what is being observed in any other region of the world [92], and that hypertension seems to be more aggressive among patients of African ancestry [93]. Losartan has been shown to be a potent, orally active, and highly selective angiotensin-II receptor antagonist for the treatment of hypertension [94]. *In vitro* and *in vivo* investigations suggest that genetic polymorphisms in *CYP2C9, CYP11B2*, *CYP3A4*, and *CYP3A5*, that affect catalytic activity of the respective enzymes, also affect response to losartan [95,96]. For example, diastolic and systolic blood pressure in patients with the CYP2C9*1/*1 genotype who were on losartan was reduced compared to the baseline level [97] in comparison to patients with other genotypes made up of deficient alleles (e.g., CYP2C9*1/*3). Since traditional herbal medicine forms part of the larger primary health system in poor countries, it is of importance that the effects of the various herbal mixtures in the different African regions be investigated for the effects of their expressions and activity of the associated DMEs. Understanding the modes of interaction between the different drugs is likely to lead to improved use of both traditional and conventional medicine.

### 3.5. Tuberculosis and Bleeding Disorders

#### 3.5.1. Herbal Management of Tuberculosis and Bleeding Disorders

Tuberculosis (TB) is a major public health concern with over 2 billion people currently infected, 8.6 million new cases per year, and more than 1.3 million deaths annually [98]. Among African traditional herbal medicine, tuberculosis is treated through targeting symptoms (for example, coughing, loss of weight and appetite, and night sweats). In the advent of resistance by the causative agent, *Mycobacterium tuberculosis* [99], the use of herbal medicine for tuberculosis treatment presents another potential means of combating the disease. Herbal medicines for the treatment and management of tuberculosis have been in existence for a long time, with traditional healers normally treating the signs and symptoms and in so doing helping to “cure” patients.

Tuberculosis (TB) is a condition that commonly occurs with HIV infection and in Southern Africa *Artemisia afra*, *Eucomis pallidiflora*, *Myrothamnus* *flabellifolius*, *Lippia javanica*, *Hypoxis hemerocallidea*, *Allium sativum*, and *Carica* papaya have been mentioned by traditional healers as some of the most regularly used herbal plants to treat and manage TB, possibly through having been shown to possess antimycobacterial activities [100]. Mesembryanthemum *edule* (also referred to as *Carpobrotus edulis*) is also one of the noted herbal plants used to treat TB infection in HIV patients among the Xhosa speaking people of the Eastern Cape in South Africa [101]. In Ghana, traditional methods of treatments based on medicinal plants are still an important part of social life and culture, and the acceptability of herbal plants as effective remedies is quite high; the most commonly used herbal plants for the management of TB include *Allium sativum*, *Azadirachta indica*, *Solanum torvum*, *Zingiber officinale*, *Phyllanthus fraternus*, *Aloe vera*, and *Cocos nucifera* [102] with a few studies showing the activity of these herbal plants against the slow-growing pathogenic strain of Mycobacterium tuberculosis [98]. It is no secret that traditional health systems have long been involved in the symptomatic treatments of tuberculosis, and whether they are effective is an argument to be taken in another review. What is clear is that the metabolism of the indicated herbal compounds or mixtures has not been a focus for studies or, alternatively, nor has the effects of these herbals on the indicated genes and their product. Thus, we highlight in this review that conventional and traditional herbal medicines will always co-exist, thus, it is our duty (researchers and health practitioners from both systems) to evaluate and understand the interactions of these drugs from both these health systems.

Bleeding disorders are also a common feature of attention among herbal medicinal practitioners, as injuries are a common occurrence in any active population. In Ghana, selected herbal plants, such as *Moringa oleifera*, *Terminalia avicennioides*, *Parkia biglobosa*, *Tapinanthus bangwensis*, and *Ficus gnaphalocarpa* are used by traditional healers for treating a number of bleeding-related disorders [38]. Antiplatelet and anticoagulant properties have been attributed to plants such as *Achillea falcata*, *Rhus verniciflua*, *Umbilicaria esculenta*, *Curcuma longa*, *and Artemisia dracunculus*, which are used ethno-medicinally in the treatment of hemorrhaging or as anticoagulants and bleeding disorders in South Africa [103,104]. In Zimbabwe, *Lannea discolour*, *Searsia tenuinervis*, *Brachylaena discolour*, *Piliostigma thonningii*, and *Pterocarpus angolensis* are some of the common herbal medicinal plants that are used for treatment and management of different bleeding disorders [105]. However, the effects of these herbals on gene expression (especially for genes of pharmacogenomics importance) have not been evaluated. Nor, are the effects of the various genes on the herbal drugs/mixtures. This review contributes to bringing such awareness and possibly paving a way for reconsideration of effectiveness of different drug systems.

#### 3.5.2. Commonly Used Drugs to Combat Tuberculosis and Bleeding and Their Pharmacogenetic Outlook

Among the several drugs used to treat tuberculosis, pharmacogenomics is known to be prominent in the disposition of isoniazid, a key first-line drug for tuberculosis treatment, with minimal pharmacogenomics indications for rifampicin [106]. Isoniazid is metabolized by N-acetyltransferase 2 (NAT2), an enzyme coded by a polymorphic gene that presents with several variants, most of which are associated with decreased NAT2 activity [107]. The most common variant alleles include NAT2*5, NAT2*6, NAT2*7, and NAT2*14, which account for the majority of the slow acetylator genotypes and have been reported across most world populations [108], however, still showing differences among populations. The slow acetylator status of NAT2 has been strongly associated with isoniazid-induced hepatotoxicity, thus, haplotypes coding for slow acetylator status could serve as useful biomarkers for prediction of anti-tuberculous drug-induced toxicity [109]. In addition to NAT2, it appears that CYP2E1 and solute carrier organic anion transporter family member 1B1(OATP1B1) coded for by *SLCO1B1* gene, could also be important biomarkers for isoniazid-induced liver toxicity in adult patients with tuberculosis [110]. For example, among Indian pediatric patients the risk of liver toxicity was enhanced by a variant in the *CYP2E1* gene [111], while the *SLCO1B1* *rs4149032* polymorphism was associated with low-level rifampin exposure [106].

Warfarin is the most commonly used oral anti-coagulant; whereas the most commonly used anti-platelet medications include aspirin and clopidogrel, each of which influences blood haemostasis through different mechanisms. Marked inter-individual variations in response to these commonly prescribed medications have been well-documented and represent a significant challenge to medical practice [112]. Warfarin consists of both R and S enantiomers, with the S being more active. Each form, however, is metabolized through a different mechanism, with the S-warfarin metabolized primarily by CYP2C9 and R-warfarin metabolized predominantly by CYP3A4 [113]. Studies suggest that genetic variation in CYP2C9 impairs metabolism of S-warfarin by about 30%–40% [114]. The distribution of *CYP2C9* polymorphisms among sub-Saharan populations is characterized by the absence or rarity (< 1%) of the defective alleles CYP2C9*2 and CYP2C9*3, which are relatively common in Europeans [115]. In addition to CYP2C9, VKORC1 and CYP4F2 also play important roles in warfarin metabolism, thus, genetic variation in their genes would result in variability in warfarin response. 

### 3.6. The Combined Use of Conventional and Herbal Medicine and their Interaction with Polymorphisms in Pharmacokinetic and Pharmacodynamic Genes

Pharmacogenetics describes patients’ variation in response to therapy due to genetic factors [18] and this is of special interest for drugs with narrow therapeutic indices, where impairment in metabolic activity is likely to cause adverse drug reactions (ADRs). The majority of phase I and phase II drug-metabolizing enzymes (DMEs) are polymorphic. Genetic polymorphism of DMEs encompasses gene copy number variation including gene amplification and deletion, small insertions and deletions, as well as single-nucleotide polymorphisms (SNPs) [10]. The polymorphisms of DME genes are important determinants for drug response, and, indeed, the majority of pharmacogenomics drug labels refer to genes encoding phase I and phase II enzymes [116]. African populations have a larger genetic diversity than other populations [117]. Genotyping of Zimbabweans [118] and Ethiopians [119] showed the existence of qualitative and quantitative differences in genetic polymorphisms in different populations.

It has been increasingly acknowledged that herbal medicines and conventional drugs can potentially interact in the same manner observed among conventional drugs (*i.e.*, drug/drug interactions) [120]. As alluded to earlier, both herbal medicinal drugs and conventional drugs are effectively removed from the human body through metabolism by the same enzyme metabolizing systems, which to a large extent involves cytochrome P450 enzymes (CYP), glutathione S-transferases (GST), UGTs, sulfotransferases, the microsomal epoxide enzyme system, and the various transporters. Thus, when one looks at populations in health transition, there are many challenges which include drug-drug, drug-herb and herb-herb interactions. One example of herb-drug interaction is the observed effects of the Ayurvedic syrup, “Shankhapushpi”, as reported by Bateman *et al.* [121], which causes a decrease in the plasma concentration of phenytoin resulting in loss of seizure control in epileptic patients. Another challenge is that there seems to be no consideration in herbal medicine dosing which takes into account the diversity of patients in terms of their being adults, children, or elderly; however, physiological differences among these groups are known.

In developing nations, traditional herbal medicine is the major source of health care for most except a privileged few [122]. There is remarkably little correlation and scientific evidence to support the safe or effective combination of traditional and conventional medical care approaches. Many people who today choose herbal products in addition to prescription medications assume that because these products are natural, they must be safe, even when the evidence for this assertion is essentially anecdotal. Studies have shown that herbal medicines are highly variable in quality and composition, with many marketed products “in some cases” containing little of the intended ingredients and actually including some unintended contaminants such as heavy metals and traces of prescription drugs [123]. Sometimes there are problems with identification of plants; for example, some plants might look similar in appearance, yet are different with respect to their herbal medicinal use. A survey in Ghana [40] reported on possibilities of misidentification of plants by some merchants. It is important, therefore, that a registry of pharmacopoeia be kept to authenticate the identity of herbal plants used by practitioners, especially formulations that claim to contain certain plant species. However, this should not distract from the fact that herbal medicines have had many centuries of use and validation among communities.

Similar to conventional drugs, herbal medicines can also induce or inhibit the expression of xenobiotic metabolizing enzymes (XMEs), thereby compromising the metabolizing of other herbals or conventional drugs that they are co-administered with. For example, it has been observed that St. John’s wort (*Hypericum perforatum*) affects the metabolism of nearly 50 percent of all prescription drugs [124] through its interaction with the drug efflux transporter p-glycoprotein (P-gp) coded for by *ABCB1* gene. *Hypericum perforatum* inhibits P-gp, leading to overdosing with P-gp substrate drugs such as fexofenadine [125]. *Hypericum perforatum* has also been shown to cause a decrease in the bioavailability of theophylline [126] when co-administered. Other herbal medicines such as *Cassia auriculata* and *Cardiospermum halicacabum* have been shown to influence the bioavailability of both carbamazepine and theophylline [127]. *Zingiber officinale* (ginger) is a potent inhibitor of thromboxane synthetase and thus prolongs bleeding time [128] and therefore influences the therapeutic outcome for persons taking warfarin or other drugs that affect platelet activity. Inhibition of coagulation leading to postoperative bleeding and requiring surgical re-exploration after preoperative use of the herbs ginkgo, ginseng, and the Chinese herb *Huang qi,* combined with vitamin E and the prescription drugs quinine sulphate and sertraline hydrochloride was reported in a 60-year-old woman with breast carcinoma [129].

### 3.7. Contestations in Knowledge Production: Issues in the Co-Existence of Conventional and Herbal Medicines

It should be acknowledged that the two health systems, conventional and traditional medicine, will co-exist for a very long time to come and continue to be used concomitantly. Some of the reasons for the above assertion include the inaccessibility, expensiveness, and ineffectiveness of conventional drugs to completely cure existing diseases (e.g., HIV/AIDS, asthma, psychotic disorders) [130]. Also, as long as traditional medicines remain cheap and retain some visible evidence of usefulness, people will always use them. Lastly, the provision of herbal medicine is cultural, and is viewed as a holistic way of healing that cures not just the ailment but also addresses spiritual issues anchored on a people’s belief system [131]. In some cases, if one is cured, it is thought to extend to members of the family and the community at large. This contrasts with conventional medicine, which is individualistic and reductive in practice.

Africa presents a diverse collection of herbal medicines reflecting the geographical and cultural diversity on the continent. As an emerging realization, a few *in vitro* studies have been done in evaluating commonly used herbal plants in Southern and West African countries on their induction or inhibition properties for drug metabolizing enzymes, as seen in Table 1. The data shows that the concurrent use of these herbal drugs with conventional medications that are metabolized by drug metabolizing enzymes, especially the cytochrome P450 enzymes, should be avoided to prevent the risk of adverse drug reactions.

One area of contention in terms of studying the effects of drugs and herbal medicines is the issue of validation [132]. In many parts of the world, traditional herbal medicine is always “accused” of toxicity even without any confirmation of that. However, when one interacts with herbal traditional practitioners, it is obvious that there are ways of administering herbal medicines that, while avoiding toxicity, achieve effectiveness. In fact, some practitioners even warn their patients on how to avoid overdosing. The major issues with herbal medicines include (1) the form in which the herbal is taken (extracts, raw, or processed), (2) quantities to be given, (3) varying effects depending on part of plant used (*i.e.*, roots, leaves, flowers, bark, or seeds), and (4) harvesting season and time of day [132,133]. However, harvesting seems to be taken into account in traditional herbal medicine through “agreed” times of collection for certain herbals. Recently, however, traditional herbal medicine has started to be exploited by some unscrupulous practitioners who enter into this health system for money. For example, in South Africa removal of unwanted pregnancies using herbal medicine is a thriving business, while in other parts of Africa “fake” herbal medicine is used to con the poor with promises of enhancing their employment prospects. All this gives traditional medicine a bad name that makes it difficult to promote the good aspects; thus, it is imperative to put in place measures to control and manage this health system. This is not without precedence: traditional Chinese medicine which emphasizes holistic aspects of healing is widely accepted world-wide, and has become a billion-dollar industry [134]. One of the problems that may have affected acceptance of herbal medicines in Africa is a history of colonialism, and Christianity, where herbal medicine was challenged as unscientific and from a dark age.

There has been a slow amalgamation of conventional and traditional herbal medicine, and mainly a looking-down upon of herbal medicines, because modern medicine comes out with a western worldview dominated by laboratory science as the dominant way of knowledge production. Traditional medicine is viewed as backward and as such in need of purification, “cleansing”, or sanitization in the laboratory for it to be safe. And yet, many generations have been using these medicines, in context-specific ways that attest to their effectiveness. It has been shown in some instances that the reductionist laboratory or western view has failed to harness activities observed in some herbal mixtures through purification [132] and needing to identify an active component. Could it be that some herbal mixtures provide a holistic amount of chemicals leading to effectiveness which is lost when evaluated in isolation? Or, can it be that some active components of herbal medicines are present in insufficient quantities in some plants, requiring some mixtures as part of boosting them? Only more research can tell, but these questions are posed to generate discussion and possible angles for evaluation of both herbal and conventional medicines.

Lastly, we beg to ask, why is it that in terms of nutrition, there is a huge obvious call for people to revert back to traditional foods (mostly unprocessed), yet this seems to exclude traditional herbal medicines? Pharmacogenomics knowledge evaluation does not assume any one of the health systems, conventional or traditional, is superior or inferior, but that, since they are affected by the same enzyme system or they affect the same enzyme system, it is of paramount importance to tease out which combinations should be avoided. We have a different opinion to the general perception that traditional African medicine is inferior and that it must only be used after scientific tests in the laboratory, as this has implications for those people without access to western or modern facilities who rely heavily on traditional herbal medicine. We do, however, accept that traditional herbal medicine is diverse and can benefit from borrowing procedures in conventional drug development to make it optimally usable, safe, and environmentally caring. There is nothing wrong with establishing regulated pharmacies that dispense traditional medicine. Thus, proper value addition to African herbal medicines is likely to lead to their acceptance, just as with Chinese traditional medicine.

It is important to acknowledge that when one talks about traditional and modern medicine, one is referring to knowledge production and its control. Using laboratory-controlled research by scientists helps in standardization of procedures and productions. Similarly, it is important that traditional practitioners embrace a code of conduct for the use of herbal medicines with an ultimate view of standardizing treatment of patients [132]. Conventional medicines that have evolved from traditional herbal medicines mostly identify single active ingredients, and this at times creates an expectation that all herbal medicines could produce identifiable, isolatable, and quantifiable active components. This may not be possible for all herbal medicines as compounds could have synergistic and/or complementary functions. It is acknowledged that both traditional and conventional medicine can be used for effective treatment of health conditions, but for a population in health transition where access to conventional medicines is limited, it becomes important to also integrate proven knowledge skills and benefits of herbal medicines into the modern systems of medicine, which can be unlocked through more research, ensuring appropriate quantities, and efficacious and safe treatment. This assists towards the establishment of appropriate guidelines for monitoring its use and side effects as done for conventional medicines. The evolution of better-equipped pharmacies for traditional medicines with appropriate manuals for consultation will improve the values and judicious use of traditional medication.

## 4. Conclusion and Outlook

This article examined similarities in the use of allopathic and herbal medicines in African populations undergoing transitions in health, with malaria, HIV, hypertension, TB, and bleeding disorders being used as examples. The review also aimed to stimulate discussion on the co-administration of these medicines with their attendant challenges. Herbal medicines are often administered in combination with therapeutic drugs, raising the potential of herb-drug interactions which leads to ADRs [39,42]. There is a lack of understanding as to how herbal medicines affect drug metabolizing enzymes or how drug metabolizing enzymes affect these herbal medicines. We have gone beyond asking the questions “Which one should be used?” or “Which one is better?” to the current question “How can we optimally make use of both in a population that has access and is using both systems?”, as it is known that both allopathic and herbal medicines are used concurrently in populations in health-transition.

For Africa, with its multiple health challenges, including constrained access to allopathic medication, the acknowledgement by the WHO in 2008 that herbal traditional medicine may play a more important role, was very relevant. The fact that literature searches yielded few results on possible herb-drug interactions highlights the paucity of research in this important area of research (Table 2). There is need for concerted research to understand the similarities in herbal medicine and allopathic medicine for treatment of the same ailments. Genetic differences among African populations with respect to genes coding for pharmacokinetic and pharmacodynamics targets affect the disposition of both allopathic drugs and traditional herbal medicines. These differences then reflect as variations in response to herbal or allopathic drug treatment for any condition, especially the commonly-occurring diseases. Medicinal herbal plants contain a lot of compounds in one mixture that pose a challenge on how they are studied (in the laboratory) with respect to interaction with drug metabolizing enzymes and allopathic medicines. There could be a need for standardization in the preparation of the herbal medicinal drugs, reflecting on the different factors that affect safety or efficacy, as mentioned earlier (e.g., parts of plant or route of administration and time of harvesting). However, this may also be a call for a paradigm shift in terms of how to study such systems. Perhaps a reductionist approach is not necessary, but the laboratory approach should be integrated with traditional herbal medicine. A focus on pharmacogenomics helps us break the wall imposed between allopathic drugs and traditional herbal medicines, thereby focusing on their metabolism instead, and on critical enzymes such as the cytochrome P450 family enzymes (Table 2). This cautions patients, physicians, and traditional medical practitioners on the use of interacting combinations which should be avoided to reduce drug-induced health problems. The effects of co-administration of these drugs include possible inhibitory and inductive effects. Thus, to add value, one needs to conduct studies to understand the modes of interaction of herbal medicines and allopathic drugs, taking into consideration the genetic variability of the African population.

**Table 2:** Commonly used herbal plants in Southern and West African countries that interact with drug metabolizing enzymes (DMEs).

Medicinal Plant	Purported Medicinal Value	DMEs Affected	Ref.
*Sutherlandia frutescens*	Natural immune booster and anti-oxidant	CYP1A2, CYP2A6, CYP2B6,CYP2C8,CYP2C9, CYP2C19, CYP2D6	[13] [135]
*Hypoxis hemerocallidea*	Natural immune booster, anti-inflammatory	CYP2E1, CYP3A4/5, CYP3A4,CYP3A5,CYP19, P-gp	[135] [136]
*Phyllanthus amarus*	Anti-cancer, anti-hepatitis, anti-HIV, anti-malarial, anti-tumour	CYP1A2, CYP2C9, CYP3A4, CYP2D6, GSTA1-1, GSTM1-1, GSTP1-1	[137] [138]
*Cassia siamea*	Anti-oxidant, anti-tumor, antimalarial	CYP2C9, GSTM1-1, GSTP1-1	[138]
*Momordica charantia*	Anti-viral, anti-mutagenic, antidiabetic, anti-inflammatory anticancer, analgesic	CYP2C9, GSTA1-1, GSTM1-1, P-gp	[138] [139] [140]
*Cassia alata*	Anti-inflammatory, anti-microbial, anti-platelet aggregation, anti-diabetic, anti-hypertensive, anti-malarial	CYP1A2, CYP2C9, CYP3A4, CYP2D6, GSTM1-1, GSTP1-1	[137] [139]
*Tridax procumbens*	Anti-bacterial, anti-protozoal, wound healing	CYP1A2, GSTM1-1	[139]
*Lactuca taraxicifoli*	Antioxidant, anti-inflammatory	CYP1A2, CYP2C9	[137]

DME = drug metabolizing enzyme.

It is important to note that although herbal medicines have been used for centuries, and that they were found to be of benefit despite some complications, there is the need to understand their role in populations that have access to both systems. It is imperative that a more systematic approach to herbal medicine is taken by health ministries of countries utilizing herbal medicine in health care so that their use can be monitored, controlled, and studied along the lines of pharmacovigilance, as in conventional medicine. However, in African populations with a high burden of disease and low income among patients, the monitoring could prevent access to this primary health care resource where access to modern health care treatment is limited. It is therefore important that a systematic pharmacovigilance approach is taken to obtain reliable information on the safety and usage of herbal medicines [141,142]. Research can determine safe dosages and manuals can be developed to defray costs while providing benefits to health.
